# Ebola Response: Modeling the Risk of Heat Stress from Personal Protective Clothing

**DOI:** 10.1371/journal.pone.0143461

**Published:** 2015-11-17

**Authors:** Adam W. Potter, Julio A. Gonzalez, Xiaojiang Xu

**Affiliations:** Biophysics and Biomedical Modeling Division, United States Army Research Institute of Environmental Medicine, Natick, Massachusetts, 01760, United States of America; King Abdullah International Medical Research Center, SAUDI ARABIA

## Abstract

**Introduction:**

A significant number of healthcare workers have responded to aid in the relief and containment of the 2013 Ebola virus disease (EVD) outbreak in West Africa. Healthcare workers are required to wear personal protective clothing (PPC) to impede the transmission of the virus; however, the impermeable design and the hot humid environment lead to risk of heat stress.

**Objective:**

Provide healthcare workers quantitative modeling and analysis to aid in the prevention of heat stress while wearing PPC in West Africa.

**Methods:**

A sweating thermal manikin was used to measure the thermal (R_ct_) and evaporative resistance (R_et_) of the five currently used levels of PPC for healthcare workers in the West Africa EVD response. Mathematical methods of predicting the rise in core body temperature (T_c_) in response to clothing, activity, and environment was used to simulate different responses to PPC levels, individual body sizes, and two hot humid conditions: morning/evening (air temperature: 25°C, relative humidity: 40%, mean radiant temperature: 35°C, wind velocity: 1 m/s) and mid-day (30°C, 60%, 70°C, 1 m/s).

**Results:**

Nearly still air (0.4 m/s) measures of R_ct_ ranged from 0.18 to 0.26 m^2^ K/W and R_et_ ranged from 25.53 to 340.26 m^2^ Pa/W.

**Conclusion:**

Biophysical assessments and modeling in this study provide quantitative guidance for prevention of heat stress of healthcare workers wearing PPC responding to the EVD outbreak in West Africa.

## Introduction

The 2013 Ebola virus disease (EVD) outbreak in West Africa is the largest outbreak of EVD in recorded history. As of July 2015, EVD has been reported in over 27 thousand cases and resulted in over 11 thousand deaths. In response to this outbreak, the number of clinical workers engaged in treatment and infection control has also been significant. As of July 2015, this dense presence of healthcare workers has led to 875 healthcare workers infected and 509 deaths [1]. Personal protective clothing (PPC) is vital for protecting healthcare workers who come in contact with EVD patients. PPC components may include coverall garment, gloves, glasses, boots etc. Use of PPC can itself create significant physiological and physical stresses to wearers, in addition to impaired vision, mobility, and communication. One of the significant stresses imposed by PPC is heat stress which limits working duration, reduces performance and can be lethal itself.

**Fig 1:**
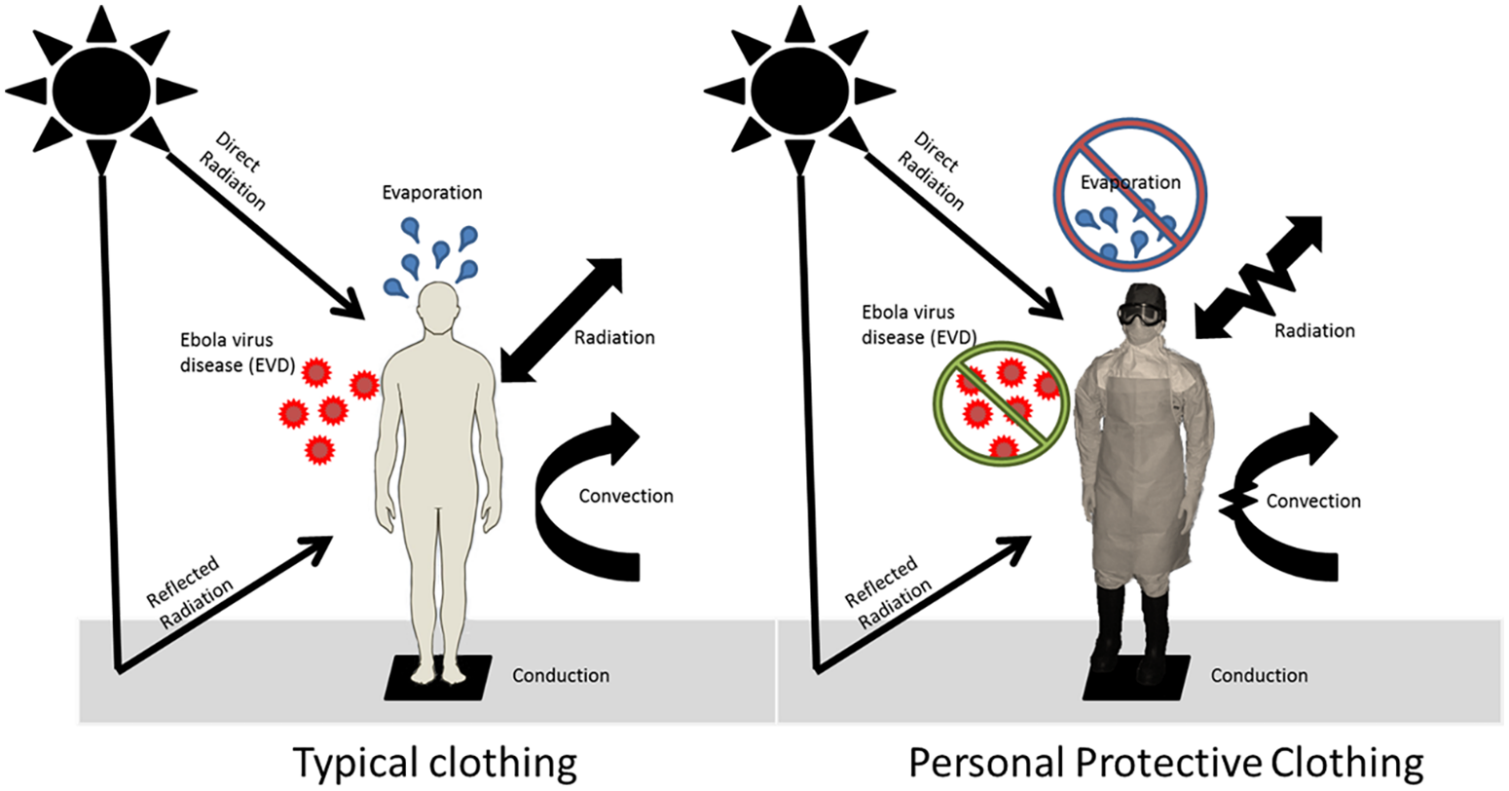
Balancing protection and heat strain: Typical non-protective clothing allows for thermoregulation and heat dissipation; while protective clothing impedes heat exchange to the environment.

Healthcare workers wear different levels of PPC that protects individuals from contracting EVD by restricting vapor molecule transfer from the environment to the body. However, this vapor impermeability and encapsulating design also restricts evaporation and general heat exchange into out to the environment increasing an individual’s risk of overheating (Fig 1). The human body generates heat which ranges from ~100W at rest to 500W or higher during working, depend on the activity. To maintain homeostasis and avoid heat injury and illness, the body must dissipate most of this heat to environment via evaporation, convection, and conduction. However, PPC increases the thermal resistance and vapor resistance, reducing heat loss from the body to the environment. The risk of heat injury becomes more severe in warm or hot environments where sweat evaporation is the only one avenue for heat loss. Wearing PPC in the hot humid conditions of West Africa puts healthcare workers at significant risk of heat stress. Heat stress results from a combination of environmental conditions, metabolic heat production, and biophysical properties of clothing [2]. The impermeable or semi-impermeable design of PPC shields the wearer from chemical and biological hazards but significantly increases risk of heat stress limiting evaporative heat transfer from the body into the environment [3–6]. Heat strain increases the risk of heat injury or illness and reduces work capacity, requiring increased demand for work-rest cycling. This work-rest demand is particularly problematic in EVD health workers as they are subject to frequent donning and doffing of PPC, adding to risk of infection [7–9].

This paper defines the biophysical properties of PPC worn, and predicts heat stress by rise in core body temperature (T_c_) to enable estimates of safe work times for health workers responding to the EVD outbreak in West Africa.

## Materials and Methods

### Personal Protective Clothing

**Fig 2:**
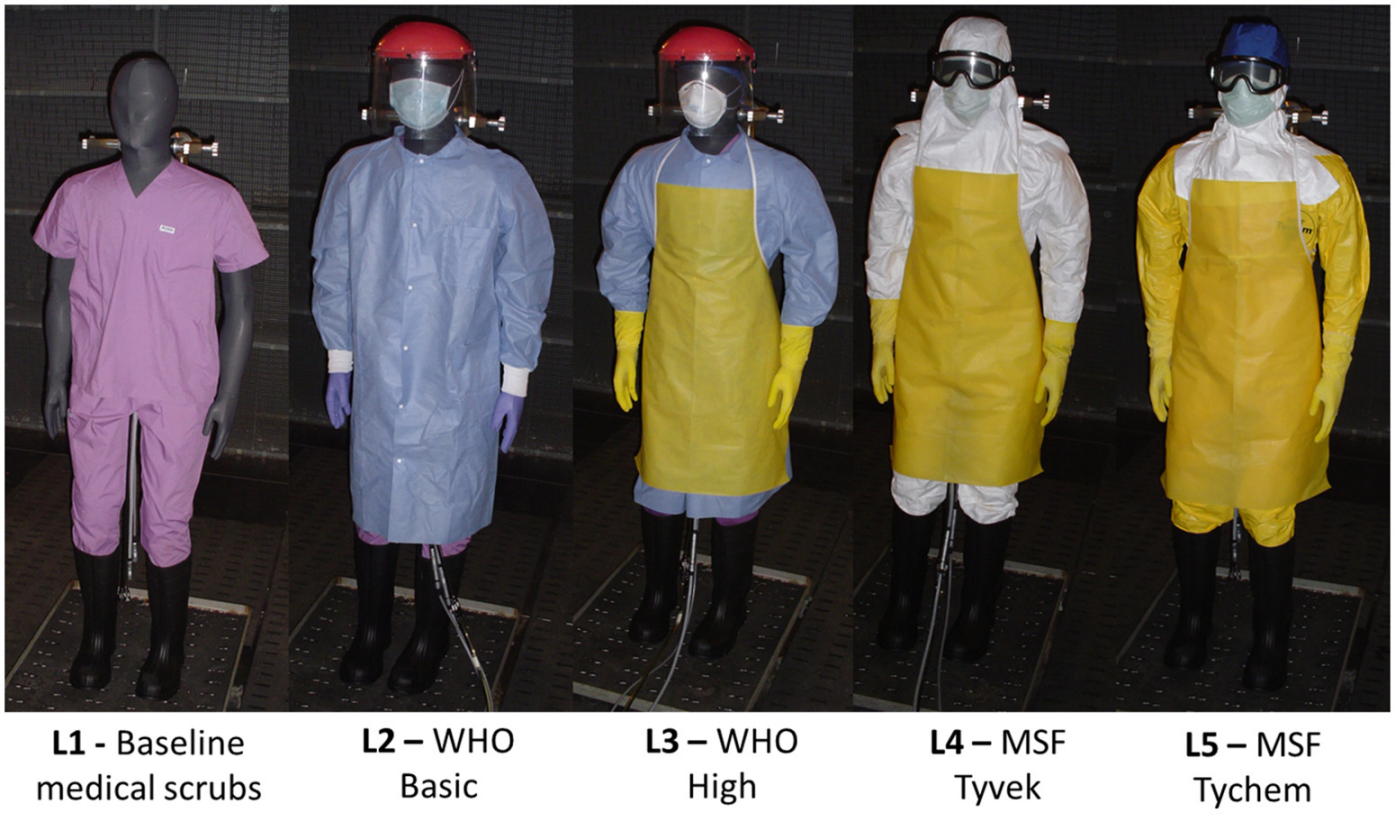
Five currently used levels of personal protective clothing by healthcare workers in the Ebola outbreak in West Africa.

Five levels of currently used PPC were assessed; three from the World Health Organization (WHO) and two from Médecins Sans Frontières or Doctors Without Borders (MSF) (Fig 2). Each configuration included a baseline layer (L1), consisting of medical scrubs with cotton socks and boxer briefs, and knee high black rubber boots. WHO Basic (L2) consists of L1 with an isolation gown, a cotton surgeon’s cap, surgical mask, nitrile examination gloves, and plastic face shield. WHO High (L3) consists of L1 and L2, apron, a N95 filtering respirator, and heavy duty long cuff gloves. MSF Tyvek (L4) consists of L1, Tyvek coverall and custom-made surgical hood, N95 filtering respirator, apron, and goggles. MSF Tychem (L5) consists of a L1, Tychem coveralls, and the same additional components as L4.

### Biophysical Assessments

Each level of PPC was tested in a climate-controlled wind tunnel, using a sweating thermal manikin (STM) (Newton 20 zone, Measurement Technologies Northwest, Seattle, WA). The STM consists of 20 independently controlled zones that are heated to simulate regional metabolic heat production and sweating. Testing was conducted according to American Society for Testing Materials (ASTM) international standards for thermal resistance (R_ct_, m^2^K/W) [10] and evaporative resistance (R_et_, m^2^Pa/W) [11]. Testing for R_ct_ quantifies dry heat exchange (convection and radiation) possible; while R_et_ testing quantifies the evaporative heat loss possible (e.g., sweating).

### Predictive Modeling

Simulations assume a generally healthy, normally hydrated (1.24% dehydrated) individual that is fully heat acclimatized. The human characteristics used thermal modeling include varied individual surface areas (A_D_), using the assumption of the relative metabolic heat production being related to 58.15 W/m^2^ [12]. Five different sizes of individual, by A_D_ (m^2^) were used to provide a range of healthcare workers; where these surface areas relate to differences in metabolic heat production at different metabolic equivalencies (METs). Three activity levels were modeled based on similar categories described by Ainsworth et al. [13]: 2 MET nursing / patient care, 3 MET walking or moderate work, and 5 MET vigorous or intermittent heavy work (Table 1).

**Table 1:** Individual sizes modeled and associated metabolic rates and MET.

Size	Surface Area (A_D_; m^2^)	2 MET (W)	3 MET (W)	5 MET (W)
**S1**	1.6	186	279	465
**S2**	1.7	198	297	494
**S3**	1.8	209	314	523
**S4**	1.9	221	331	552
**S5**	2.0	233	349	582

Mathematical methods from Giovoni and Goldman [14] were used to estimate rise in core body temperature (T_c_). Specific inputs for this modeling process requires measures of the clothing biophysical characteristics (R_ct_ and R_et_), environmental conditions (T_a_, RH, T_mr_, V_w_), activity (METs), and individual characteristics (A_D_, hydration status, heat acclimatization). To enable predictions T_c_ rise, foundational estimations were calculated for the evaporation required for balancing heat (E_req_) (Eq 1), maximal evaporative capacity (E_max_) (Eq 2), and the radiative and convective heat transfer (Dry) (Eq 3), of given conditions, as:

$$E_{req}=M-W_{ex}+Dry$$ (1)

$$E_{max}=A_{D}•\frac{P_{s,sk}-RH•P_{a}}{R_{et}}$$ (2)

$$Dry=A_{D}•\frac{T_{sk}-T_{db}}{R_{ct}}$$ (3)

where M is metabolic rate, W_ex_ is external work performed, P_s,sk_ is saturated vapor pressure at the skin temperature (pascal), P_a_ air vapor pressure (pascal), T_db_ is the dry bulb temperature (°C), $\overset{-}{T}$_sk_ is the average skin temperature (°C) at the surface.

Empirically derived methods from Givoni and Goldman [14] were used, where given inputs of environment, PPC clothing, activity, and initial T_c_ (T_c,0_), a predicted rise in T_c_ can be estimated from:

$$T_{cf}=T_{c,o}+0.004•M+0.0025+0.0011•Dry+0.8•e^{0.0047•\left(E_{req}-E_{max}\right)}$$ (4)

where T_cf_ is final core temperature at equilibrium with the environment.

### Simulated Environment

From a thermophysiological perspective, weather conditions in West Africa are fairly harsh year-round; where average air temperatures (T_a_), relative humidity (RH), mean radiant temperature (T_mr_), and wind velocities (V_w_) restrict effective heat exchange. Modeled environments conditions represent typical weather year-round for three areas of West Africa: Liberia; Sierra Leone; and Guinea (obtained from www.weatherspark.com). Simulated environmental conditions (T_a_, RH, T_mr_, V_w_) used were: morning / evening (25°C, 40%, 35°C, 1 m/s) and mid-day (30°C, 60%, 70°C, 1 m/s). Mean radiant temperature (T_mr_) was calculated as an additive factor relative to T_a_, where T_a_ + 40 = 100% full sun (morning / evening T_mr_ = T_a_ + 10; mid-day T_mr_ = T_a_ + 40).

## Results

**Fig 3:**
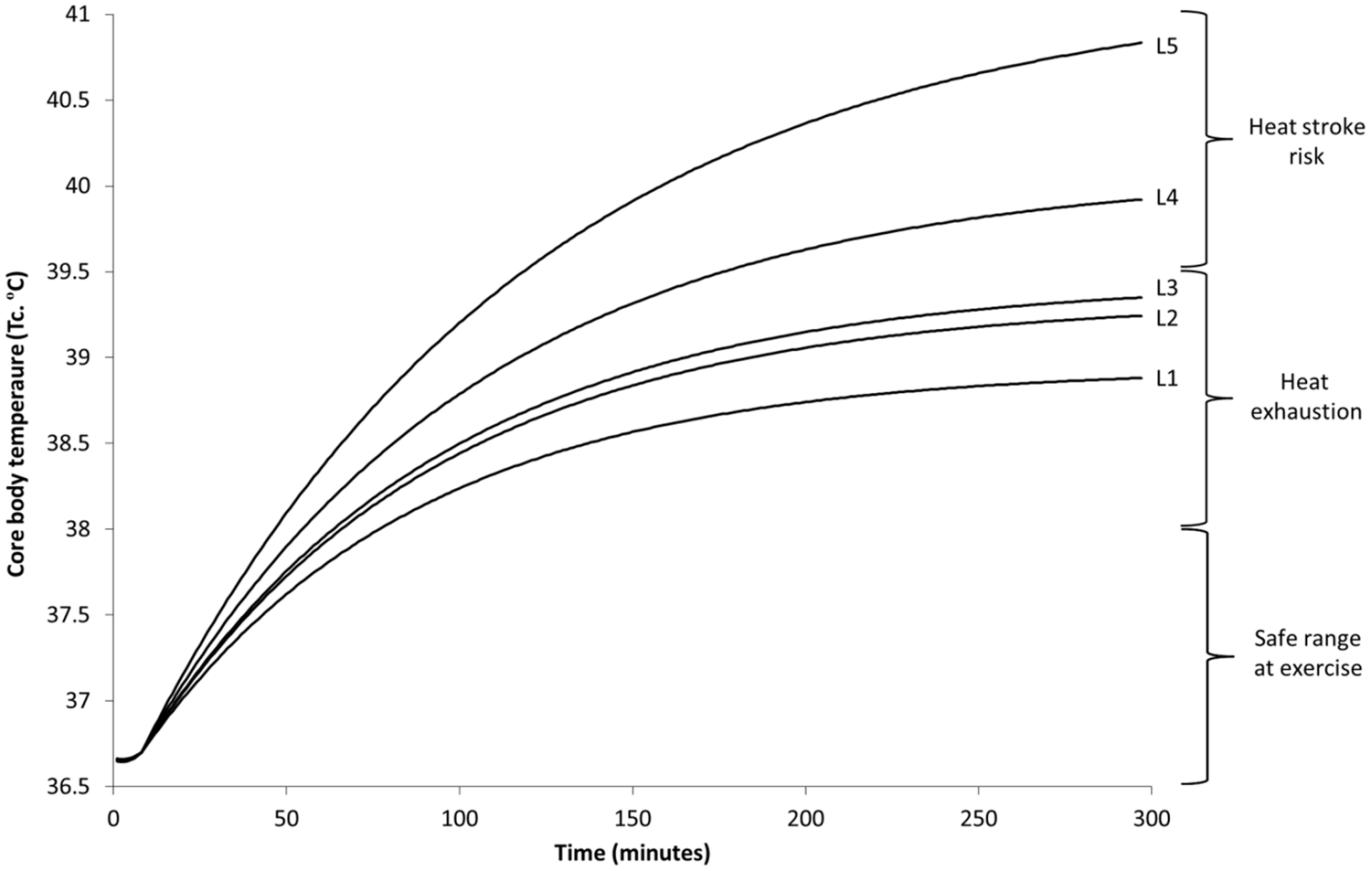
Predicted rise in core body temperature of average size person (1.8 m^2^) during hot humid conditions (30°C, 60%, 70°C, 1 m/s), working at moderate intensity (3 MET; 314W), in five different levels of personal protective clothing (L1-L5).

Biophysical testing of each of the five levels of PPC show with increasing layers of protection there are relatively no differences in insulation properties (i.e., R_ct_); while significant decreases in permeability (i.e., increased R_et_) [Table 2]. The decreased permeability significantly adds to heat strain, where increased resistance to vapor transfer directly relates to reduction in evaporative heat loss. This difference in the thermal effects of the human can be seen when the environment, activity level, and individual parameters are held constant, while varying each level of protection (Fig 3).

**Table 2:** Ensemble thermal and evaporative resistance measures at 0.4 m/s.

Configuration	Short Description	Thermal Resistance (R_ct_) (m^2^ K/W)	Evaporative Resistance (R_et_) (m^2^ Pa/W)
**L1**	Baseline medical scrubs	0.18	23.53
**L2**	WHO Basic	0.24	47.42
**L3**	WHO High	0.26	53.05
**L4**	MSF Tyvek	0.25	93.44
**L5**	MSF Tychem	0.26	340.26

**Fig 4:**
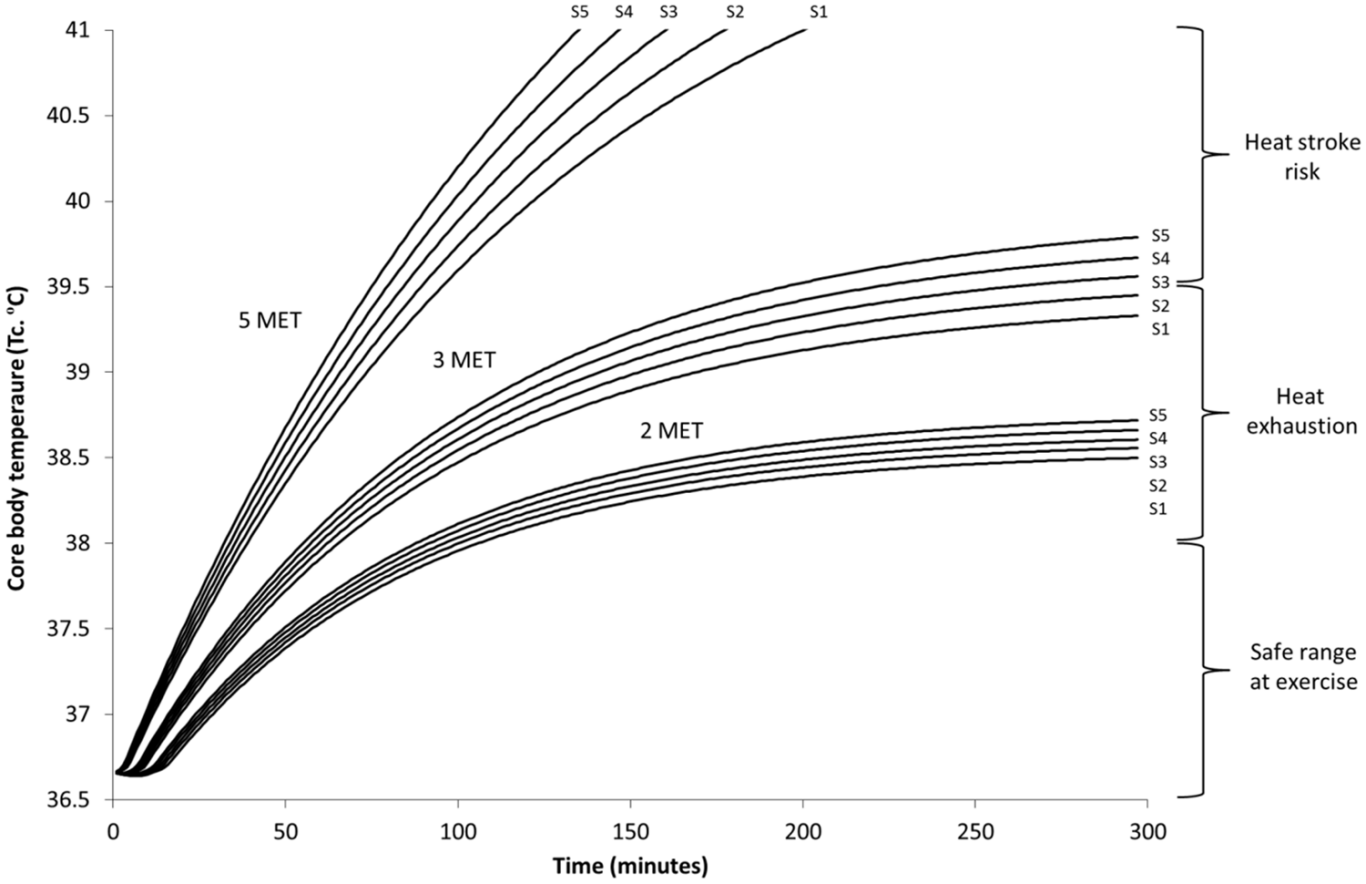
Predicted rise in core body temperature of five individual body sizes (S1-S5) (m^2^) during hot humid low solar (morning / evening) conditions (25°C, 40%, 35°C, 1 m/s), working at three different intensities (2, 3, and 5 MET), wearing the highest level of personal protective clothing (MSF Tychem; L5).

**Fig 5:**
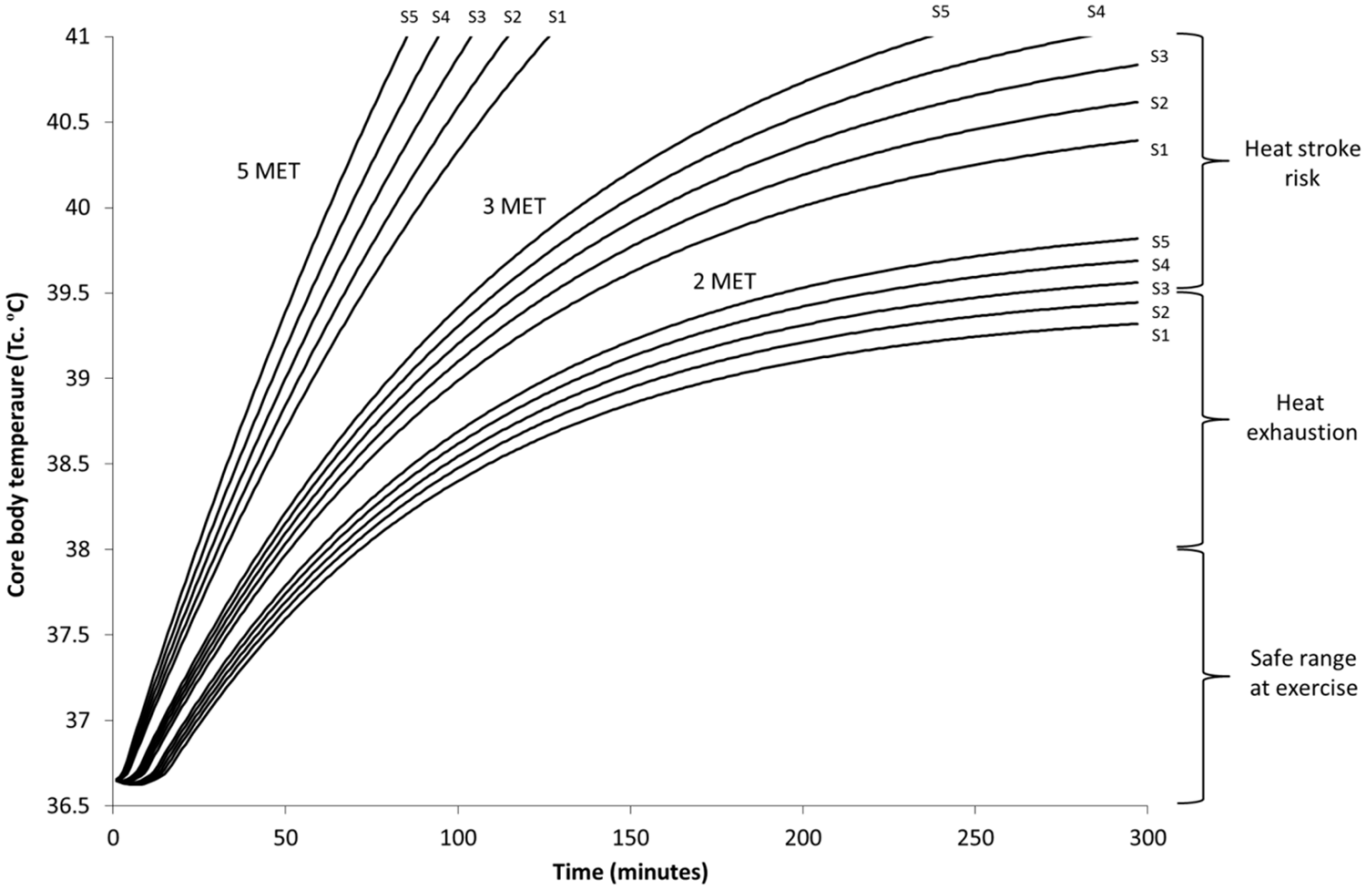
Predicted rise in core body temperature of five individual body sizes (S1-S5) (m^2^) during hot humid high solar (mid-day) conditions (30°C, 60%, 70°C, 1 m/s), working at three different intensities (2, 3, and 5 MET), wearing the highest level of personal protective clothing (MSF Tychem; L5).

Increases in body size and associated metabolic heat production add to the risk of heat stress on individuals and becomes increasingly problematic at higher intensity work (Fig 4), at peak solar hours of the day (Fig 5), and when wearing increasing levels of protection (Fig 3).

Duration of safe working times can be estimated by using reference limits; where 38.0°C can be seen as a safe limit for the general population [12], while critical upper limits between 38.6–39.5°C can be used for uncompensable conditions [15]. Clinical T_c_ reference points define heat exhaustion between 37–40°C and heat stroke above 40°C when accompanied with central nervous system irregularities [16].

## Discussion

This is the first study that directly assesses quantifying heat stress imposed on healthcare workers responding the 2013 EVD outbreak. The study measured the specific biophysical properties, i.e., R_ct_ and R_et_, of the currently used PPC in the EVD response. This study also modeled rise in T_c_ based on various levels of PPC, individual size differences, and by low and high solar conditions. The increases layers of PPC, individual size, and added solar load directly increase the level of heat stress imposed on healthcare workers and reduces the duration of safe working times.

Working time could be managed with effective use of work/rest cycles, where based on level of PPC, individual characteristics, activity level, and environment, a ratio of work time (i.e., in PPC) and resting time (i.e., time out of the suit and/or within a cooler environment) could be applied. Determining a definitive guidance for work/rest cycle management is difficult due to the complex interaction between each of the key elements (i.e., human characteristics, clothing, environment) during both the activity and the rest component. However, using results of this paper, specifically Figs 4 and 5, and guidance outlined by U.S. Army TB-MED 507 [17], a general guideline could be applied for determining this ratio. The environmental conditions and impermeable conditions from PPC lower the core temperature limits and as such, work/rest should be managed with this in mind. Modeled by hour guidance is outlined in Table 3 for work over a four hour period, where modeling assumes rest points occur when individuals reach ~37.8°C and over the course of the four hour period does not rise above ~38.5°C. While all elements (e.g., individual acclimation, hydration) need to be considered in practice; Table 3 can be used as a simplified guideline for work/rest based on two levels of temperature and humidity, and the different intensity activities (i.e., MET level).

**Table 3:** Modeled work/rest guidance over a four hour period for average size person (1.8 m^2^) based on activity level and environment wearing the highest level of personal protective clothing (MSF Tychem; L5).

Environmental Condition	2 MET Work/Rest (mins)	3 MET Work/Rest (mins)	5 MET Work/Rest (mins)
**25°C, 40% RH**	50 / 10	40 / 20	30 / 30
**30°C, 60% RH**	50 / 10	30 / 30	20 / 40

This study seeks to provide planning insights for guiding the prevention of heat related illnesses on the healthcare workers. The results demonstrate clearly that PPC imposes heat strain on healthcare workers and safe work times are limited under typical West Africa environmental conditions. Thus effective strategies to manage heat strain are critical to ensure that healthcare workers have adequate time to complete their tasks without heat illness. Work-rest cycles should be closely monitored to allow individuals to cool down, with sufficient time so that conscious and deliberate decisions can still be made when doffing PPC. While managing work-rest cycles is of significant importance, it is also well recognized that improvements to PPC and more active methods of cooling are needed to extend these maximal safe work times for the individual healthcare workers [18].

Active personal cooling systems (PCS), i.e., both liquid cooling system (LCS) and air cooling system (ACS), have been proven effective at reducing heat stress since the early 1970s [19–20]. These PCS can remove heat generated by the body to keep the body in heat balance state. Different methods for providing personal cooling while wearing PPC have been extensively worked on by the U.S. Army [21–26]. To date, LCS have been shown as the most efficient cooling method when used underneath PPC [27–31]. LCS consist of a liquid cooling garment (LCG, usually vest) and a cooling unit; where the LCG vest has small plastic tubes on the inside surface (tube length about 20 m or longer). When cooled water from the cooling unit circulates through the tubes, it removes heat directly from human body. ACS usually consist of an air distribution garment and a cooling unit; where the cooling unit blows cool air around the body, i.e., torso, through the liner of the air distribution garment. The moving cooling air removes heat from human body [32].

Selection of LCS or ACS is dependent on operational conditions, requirements and available resources. LCS requires cooling units which can deliver ~200W cooling continuously or portable cooling units which can deliver ~200W cooling for a limited time period. This ~200W can effectively improve work duration at high intensity (e.g., 5 MET) to the same as lower intensity activities (e.g., 3 MET) or can significantly reduce thermal strain at and improve thermal comfort at lower intensity work. Therefore, LCS is suitable to vehicle and medical high level isolation rooms in hospitals where power supply and space for the cooling units are available. Portable LCS is suitable to conditions where staff moves lots and portable cooling units can be recharged. Simple portable cooling units use ice, operate on battery and work for several hours. ACS are suitable to conditions where power supply and space for the cooling units are available, such as high level isolation rooms in hospitals. However, LCS cooing units usually are more efficient and compact in compared with ACS. Therefore, portable ice LCS are the preferable systems to alleviate heat stress association with PPC when dealing with EVD patients. The system will keep healthcare workers relatively in thermal comfort conditions and extend working duration to complete the tasks. Insights and improvements to some of these different methods can be leveraged to provide significant benefits to healthcare workers in these harsh environments, both for heat stress reduction and thermal comfort perspectives.

Manikin testing and modeling methods outlined in this paper can be used to simulate physiological responses and evaluate PPC as well as cooling methods for mitigation of heat stress. With the significant risk of heat stress imposed on healthcare workers responding to the EVD outbreak, improved PPC, cooling methods, and work-rest management are essential for protecting individuals from heat injuries. Methods from this paper represent a quantitative, economical, and time efficient means of assessing thermophysiological strain imposed while wearing PPC in hot humid environments.

## Conclusions

This study provides insight that can be used to guide safe working time durations for healthcare workers responding to the EVD outbreak in West Africa. The analysis showed that PPC imposes heat strain to healthcare workers and personal cooling systems are necessary to increase maximal safe work time. While this information is limited to being modeled to a range of specific conditions, it can be used as a general reference for many conditions not specifically outlined within this manuscript.

Modeling methods outlined in this study provide economical assessments of heat stress imposed by clothing, activities, and environments, that can be designed specifically for a given individual or population.

## Supporting Information

S1 File Measured thermal and evaporative resistances. (XLSX)
